# Medical and productivity costs after trauma

**DOI:** 10.1371/journal.pone.0227131

**Published:** 2019-12-30

**Authors:** A. J. L. M. Geraerds, Juanita A. Haagsma, L. de Munter, N. Kruithof, M. de Jongh, Suzanne Polinder

**Affiliations:** 1 Erasmus MC, University Medical Center Rotterdam, Department of Public Health, Rotterdam, The Netherlands; 2 Department Trauma TopCare, ETZ Hospital (Elisabeth-TweeSteden Ziekenhuis), Tilburg, the Netherlands; University Hospital Zurich, SWITZERLAND

## Abstract

**Background:**

Well-advised priority setting in prevention and treatment of injuries relies on detailed insight into costs of injury. This study aimed to provide a detailed overview of medical and productivity costs due to injury up to two years post-injury and compare these costs across subgroups for injury severity and age.

**Methods:**

A prospective longitudinal cohort study followed all adult (≥18 years) injury patients admitted to a hospital in Noord-Brabant, the Netherlands. Patients filled out questionnaires 1 week, 1, 3, 6, 12 and 24 months after trauma, including items on health care consumption from the medical consumption questionnaire (iMCQ) and productivity loss from the productivity cost questionnaire (PCQ). Furthermore, injury severity was defined by Injury Severity Score (ISS). Data on diagnostics was retrieved from hospital registries. We calculated medical costs, consisting of in-hospital costs and post-hospital medical costs, and productivity costs due to injury up to two years post-injury.

**Results:**

Approximately 50% (N = 4883) of registered patients provided informed consent, and 3785 filled out at least one questionnaire. In total, the average costs per patient were €12,190. In-hospital costs, post-hospital medical costs and productivity costs contributed €4810, €5110 and €5830, respectively. Total costs per patient increased with injury severity, from €7030 in ISS1-3 to €23,750 in ISS16+ and were lowest for age category 18-24y (€7980), highest for age category 85 years and over (€15,580), and fluctuated over age groups in between.

**Conclusion:**

Both medical costs and productivity costs generally increased with injury severity. Furthermore, productivity costs were found to be a large component of total costs of injury in ISS1-8 and are therefore a potentially interesting area with regard to reducing costs.

## Introduction

Injury is a leading cause of death and disability across the world, and therefore a major public health problem [1, 2]. Furthermore, injury comprises serious health care costs, posing a tremendous burden on society. In addition to health care costs, productivity loss due to injury adds up to the costs related to injury. Injuries comprise a great variety of injury types and severity levels, ranging from frequent minor injuries (e.g. superficial injuries) to rare major injuries (e.g. polytrauma). As a consequence, each injury type and severity level requires different treatment. In order to enable a well-advised priority setting of both health care provided after injury and injury prevention, it is necessary to gain insight in the costs per injury category and severity level [3]. Injury severity should be considered in a cost-analysis, as it is of great influence on the medical care that will be needed. More specifically, a patient with severe injuries will most likely need more care after hospital discharge than a patient with mild injuries. Furthermore, the type of care needed after injury affects the total costs, as specialised care is much more costly than, for example, a visit to a general practitioner (GP). However, as minor injuries are more prevalent, they can therefore result in high costs as well. Therefore, a detailed registration of both injury severity and medical care received after hospital discharge is of great relevance to gain insight in costs of injuries. Moreover, a distinction should be made between medical costs and productivity costs, in order to determine where improvement resulting in cost-control is necessary.

Previous research on costs of injury is scarce, and has mainly focussed on specific injury types rather than injury severity. Some examples of specific injury types that have been studied are traumatic brain injury [4, 5], hand injury [6] and spinal cord injury [7]. The conclusion of these studies is unanimous: the costs of the injury of interest are substantial. However, a comparison between the costs of different injury groups based on injury severity is scarce. Furthermore, extramural health care costs after discharge are often not taken into account. Some studies looked into the costs of injury in general, showing that costs of injury vary by injury type, but are in general high. However, the majority of these studies are lacking detailed injury severity and/or cost information of health care and productivity loss after hospital discharge on individual level [8–10]. One study that looked into the lifetime costs of injuries is the study by Corso et al. [11]. In this study, direct health care costs were considered as well as indirect costs such as productivity loss. However, costs were reported by injury mechanism, not by injury severity, and costs of diagnostics were not included. Two other studies on the cost of trauma focussed on the in-hospital costs and did not consider health care costs after hospital discharge [3, 12].

This study aimed to provide a detailed overview of the in-hospital and post-hospital health care costs and productivity costs due to injury up to two years after presentation at the emergency department (ED), and to provide an overview of differences in health care and productivity costs after injury for subgroups based on injury severity and age. The data that were used in this study consist of highly detailed registries of both injury severity and medical care used after hospital discharge. The highly detailed registries, including diagnostic costs during hospital stay (such as costs of a CT-scan), contribute to the uniqueness of this study, as costs can be displayed on a more detailed level than has been done in previous research.

## Methods

### 2.1. Data source

Data on injury severity and health care consumption was acquired from the Brabant Injury Outcome Surveillance (BIOS) study [13]. The BIOS study is a large prospective follow up cohort study of adult injury patients (≥18 years) in 10 participating hospitals. The study was approved by the Medical Ethics Committee of the Province of Brabant (METC-code: NL50258.028.14). Written informed consent was obtained from participants. The inclusion criteria were admission to the hospital through the ED and survival up to hospital discharge [13]. Patients were included in the study in the period from 1 August 2015 until 30 November 2016. Exclusion criteria were death within one week after hospital discharge, the absence of a permanent address of residence, the inability to understand the Dutch language, and the suspected presence of a pathological fracture [13].

If participants did not complete the first questionnaire, they were not excluded from the BIOS study but were still invited at the subsequent time points. In case a participant was incapable of filling out a questionnaire due to for example severe injuries or dementia, a proxy informant filled out the questionnaire. Data on diagnostics during hospital stay were retrieved from the hospital registries. In our study, participants from the BIOS study were included if they filled out at least one questionnaire. Participants who were registered at the ED, but failed to fill out at least one questionnaire were considered non-responders.

### 2.2. Demographics and injury characteristics

Demographic information of the patients, such as age and gender, were registered at hospital admission. Furthermore, injury severity was defined by the Injury Severity Score (ISS) [14]. The ISS is calculated with the Abbreviated Injury Scales (AIS) updated version (AIS08), which describes the type, location and severity of an injury [15]. The ISS is based on the three most severely injured body regions. The highest AIS of these three body regions are squared to calculate the ISS. ISS ranges from 1 (minor injuries) to 75 (untreatable injury). ISS were categorized in four groups: ISS 1–3, 4–8, 9–15 and 16+. Furthermore, injuries were categorized based on cause of injury: home and leisure, traffic, occupational, sport, self-harm, intentional (caused by others), and other causes that did not match any of the previous categories. Each injury was also grouped into a classification of most prevalent injuries based on the nature of the injury, which was retrieved from the AIS codes. The 15 injury groups that were used were: pelvic injury, hip fracture, and tibia fracture/complex foot fracture or distal/shaft femur fracture (lower extremity groups); shoulder and upper arm injury, and radius, ulna or hand fracture (upper extremity groups); mild traumatic brain injury (TBI) (AIS 1–2), serious TBI (AIS 3) and severe TBI (AIS ≥ 4) (head injury groups); face injury; thorax injury, and rib fracture (thorax injury groups); mild abdominal injury (AIS ≤ 2) and severe abdominal injury (AIS ≥ 3) (abdomen injury groups); spinal cord injury, and stable vertebral fracture/disc injury (spine injury groups). As some respondents sustained multiple injuries, these respondents are classified in multiple injury groups.

### 2.3. Health care use and cost calculations

Health care use was reported by patients in the follow-up surveys at 1, 3, 6, 12 and 24 months after trauma. Each of these surveys included the Medical Consumption Questionnaire (iMCQ) [16]. The iMCQ is a non-disease specific questionnaire that consists of questions related to the use of health care. Questions relate to intramural medical care (e.g. stay at hospital or other institution) and extramural health care (e.g. day treatment at an institution, homecare, visits to care providers) that was received as a consequence of the trauma. Health care use was reported for each period between two questionnaires. If respondents did not fill out the questionnaire at 1 week and 1 month, but did fill out the questionnaire at 3 months, then health care use was reported for the 3 previous months. Diagnostic activities were not reported by patients in the surveys, but were retrieved from the hospital registrations.

Total health care use per respondent was determined per period in between surveys. More specifically, all respondents of the survey at one month were included in the health care use of period one, all respondents at three months were included for the health care use between one and three months after hospital discharge, etcetera. A distinction was made between respondents with different entry moments to the follow-up (e.g. start follow-up at 1 month or start at 3 months). Since not all respondents filled out every question in the survey, the mean costs per health care item are based on the respondents that did answer the corresponding questions. Therefore, total costs cannot be based on the average costs per cost component, as the cost components comprise different groups of respondents within the included population.

Unit costs of all health care activities that were undertaken (diagnostics excepted) were retrieved from a cost-reference manual and are presented in S1 Table [17]. Unit costs of diagnostics were retrieved from hospital price lists, previous research and the NZa (Dutch health care authority)[18–25]. Total health care use was determined, and multiplied with the cost per unit. Medical costs were divided into two categories: in-hospital costs and post-hospital costs. In-hospital costs consisted of costs of: transportation to the ED, ED visit, stay at a hospital ward, stay at intensive care unit (ICU) and diagnostics. Post-hospital medical costs consisted of the costs of: stay in an institution (nursing home, rehabilitation centre or psychiatric institution), day treatment at an institution, homecare (domestic care, help with all day activities or nursing) and visits to practitioners (GP, company doctor, psychologist, social worker, physiotherapist, occupational therapist, speech therapist or dietician). The questionnaires one and two year after trauma informed on homecare, GP, company doctor, psychologist and physiotherapist only.

### 2.4. Productivity costs

Apart from medical costs, also productivity costs were included in the analyses. Productivity loss at work was reported by respondents with the iMTA productivity cost questionnaire (PCQ) [26]. Respondents reported in the PCQ on both absenteeism, absence of work due to injury, and presenteeism, being present at work after injury but being less productive than before injury. Work presenteeism was indicated on a scale from 1 to 10, with 1 being not productive at all, and 10 being at the pre-injury productivity level. Furthermore, the duration of reduced productivity was reported in number of working days. Mean costs of productivity loss were determined based on absenteeism with the friction cost method. The friction period was set at 85 days, meaning that costs were calculated up to 85 days of absence at work, as after this period a replacement is likely to have been found for the absent employee [17]. The average wage rates per gender can be found in S1 Table. In case of missing data on the average number of hours a respondent worked per week, the national mean based on gender was used: male: 36 hour per week; female: 26 hour per week [27]. By multiplying the total number of hours work missed with the hourly wage rate, the productivity loss was determined. The productivity costs were determined for the working age population (18-67y) only, as costs would be underestimated with the inclusion of elderly.

### 2.5. Data analyses

Responders and non-responders were compared to see whether responders were different from non-responders. Distribution of age, gender, ISS and length of hospital stay were compared using a chi-squared test for categorical variables, Mann-Whitney U test and independent sample T-test for continuous variables. P-values<0.05 were considered to indicate statistical significance. Subgroups of different injury severities, sex and age were compared to see whether these differed in costs. Age was divided in 18-24y, 25-44y, 45-64y, 65-74y, 75-84y and 85y+, and ISS was divided in categories ISS1-3, ISS4-8, ISS9-15 and ISS≥16+. Furthermore, an interaction term for age and injury severity was tested for statistical significance using linear regression with age, injury severity and the interaction term as explanatory variables for dependent variables: in-hospital costs (model 1), post-hospital costs (model 2), productivity costs (model 3) and total costs (model 4). All analyses were conducted in SPSS V.24 (Statistical Product and Service Solutions, Chicago, Illinois, USA).

## Results

### 3.1. Study population

**Fig 1:**
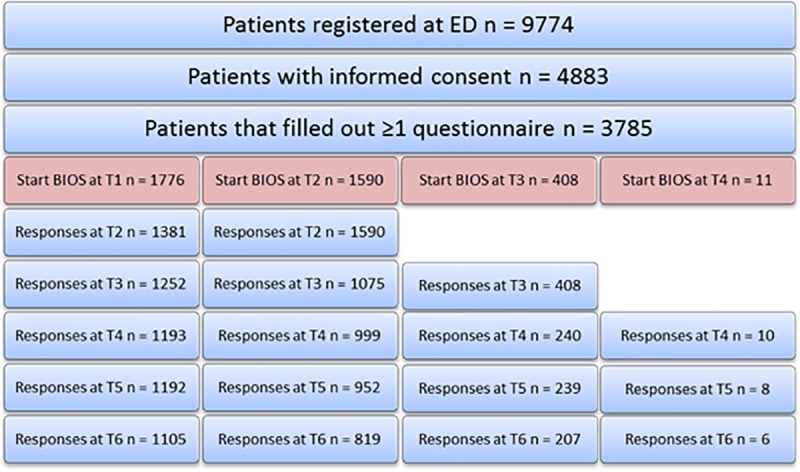
Flow chart of the research population.

A total of 4883 patients participated in the BIOS study (response rate 50.0%), and 3785 patients (77.5%) filled out at least one follow-up questionnaire on health care use and return to work and were included in the analyses (Fig 1). Approximately, half of the patients entered the study 1 week after injury (46.9%) at T1, and another 42.0% 1 month after injury (T2). The socio-demographic characteristics of the study population were summarized in Table 1, with a comparison between responders and non-responders expressed in the p-value. Even though the mean age of respondents and non-respondents was approximately the same (64.2y vs. 62.4y), the deviation over age groups differed significantly. The deviation of respondents over ISS categories differed significantly as well (p < 0.05), with the exception of ISS≥16.

**Table 1:** Characteristics of the study population divided in responders and non-responders.

Characteristic	Responders	Non-responders	p-value
***n***	3785	5989	
***Age***	64.2 (18.9)	64.4 (22.5)	< 0.001*
**18–24**	158 (4.2%)	459 (7.7%)	
**25–44**	433 (11.4%)	850 (14.2%)	
**45–64**	1127 (29.8%)	1243 (20.8%)	
**65–74**	755 (19.9%)	771 (12.9%)	
**75–84**	784 (20.7%)	1348 (22.5%)	
**≥85**	528 (13.9%)	1318 (22.0%)	
**Females**	1911 (50.5%)	3127 (52.2%)	0.097
***ISS scores***			< 0.001*
**1–3**	869 (23.0%)^a^	1636 (27.3%)^b^	
**4–8**	1248 (33.0%)^a^	1669 (27.9%)^b^	
**9–15**	1452 (38.4%)^a^	2032 (33.9%)^b^	
**16+**	187 (4.9%)^a^	246 (4.1%)^b^	
***External cause***			< 0.001*
**Home and leisure**	2213 (58.5%)^c^	3204 (53.5%)^d^	
**Traffic**	999 (26.4%)^c^	1134 (18.9%)^d^	
**Occupational**	165 (4.4%)^c^	172 (2.9%)^d^	
**Sport**	259 (6.8%)^c^	209 (3.5%)^d^	
**Self-harm**	10 (0.3%)^c^	29 (0.5%)^d^	
**Intentional (caused by others)**	45 (1.2%)^c^	160 (2.7%)^d^	
**Other**	34 (0.9%)^c^	48 (0.8%)^d^	
***Injury type*^*e*^**			
**Pelvic injury**	250 (6.6%)	194 (3.2%)	
**Hip fracture**	979 (25.9%)	1386 (23.1%)	
**Tibia, complex foot or femur fracture**	1414 (37.4%)	2009 (33.5%)	
**Shoulder and upper arm injury**	354 (9.4%)	536 (8.9%)	
**Radius, ulna or hand fracture**	243 (6.4%)	348 (5.8%)	
**Mild TBI (AIS 1–2)**	1013 (26.8%)	1754 (29.3%)	
**Serious TBI (AIS 3)**	97 (2.6%)	159 (2.7%)	
**Severe TBI (AIS≥4)**	58 (1.5%)	96 (1.6%)	
**Facial fracture**	196 (5.2%)	356 (5.9%)	
**Thoracic injury**	161 (4.3%)	199 (3.3%)	
**Rib fracture**	421 (11.1%)	518 (8.6%)	
**Mild abdominal injury**	74 (2.0%)	102 (1.7%)	
**Severe abdominal injury**	29 (0.8%)	37 (0.6%)	
**Spinal cord injury**	18 (0.5%)	19 (0.3%)	
**Stable vertebral fracture or disc injury**	238 (6.3%)	312 (5.2%)	

SD standard deviation

*Significant at 5% level

^a^29 missing values

^b^406 missing values

^c^28 missing values

^d^1017 missing values

^e^Percentages add up to more than 100% as respondents can have multiple injuries

### 3.2. In-hospital costs

The total in-hospital costs were on average €4810 per patient and were mainly driven by the costs of stay at a ward (€3070 per patient, with mean length of stay of 6 days) (Table 2; S2 Table: includes sample size and range). Average costs per person of transportation to the hospital (€720) and ICU stay (€410) were highest for the youngest age group (18-24y). The high average costs due to ICU stay in the youngest age group were due to a high percentage of ICU admittance (12.0% of 18-24y admitted to ICU with average duration of ICU stay 2.6 days). Median ICU costs were zero for all groups, except for ISS≥16 due to low percentage ICU admission in these groups. Median costs of zero in combination with an interquartile range (IQR) of [0,0] indicated that costs were made by a small percentage of the group. Average costs per person of in-hospital stay at a ward were highest for the oldest age group (85y+) (€4090). Furthermore, whereas costs of stay at ICU were higher for males (€310 vs. €140), costs of stay at a ward were on average higher for females (€2810 for male vs. €3330 for female). Mean costs per person of diagnostics were €950. Costs of diagnostics were approximately the same for males and females (€940 vs. €970). Age groups 65-74y and 75-84y had the highest costs of diagnostics (€1170 and €1170). Costs of diagnostics increased with age up to age 74y, and decreased after age 84y.

**Table 2:** Mean health care costs and productivity costs in 2017 euro per ISS category, gender and age group for responders.

		In-hospital*				**Post-hospital**				**Productivity**	**Total**
		Ambulance transport	ICU	Ward	Diagnostics	Stay in institution	Day treatment	Homecare	Practitioner visit	Productivity costs	Total costs
**Total**	Mean	620	220	3070	950	1140	430	3070	1050	5830	12190
	Median	690	0	1812	649	0	0	0	407	2654	6570
	[IQR]	[690, 690]	[0, 0]	[906, 3624]	[357, 1143]	[0, 0]	[0, 0]	[0, 495]	[52, 1368]	[0, 10078]	[3551,14786]
***Age***											
18–24	Mean	720	410	1920	520	230	400	460	890	4940	7980
	Median	690	0	906	383	0	0	0	170	2399	3844
	[IQR]	[347, 690]	[0, 0]	[453, 1359]	[225, 687]	[0, 0]	[0, 0]	[0, 0]	[0, 901]	[165, 8209]	[1916, 11301]
25–44	Mean	650	310	2230	640	420	640	760	1030	7830	12310
	Median	690	0	1359	495	0	0	0	325	5792	7360
	[IQR]	[690, 690]	[0, 0]	[906, 2265]	[279, 832]	[0, 0]	[0, 0]	[0, 0]	[60, 1274]	[1596, 13460]	[3145, 16802]
45–64	Mean	610	260	2570	860	470	530	1000	1230	6410	12650
	Median	690	0	1359	567	0	0	0	572	3691	8285
	[IQR]	[690, 690]	[0, 0]	[906, 2718]	[320, 1000]	[0, 0]	[0, 0]	[0, 0]	[102, 1663]	[0, 11047]	[3694, 17363]
65–74	Mean	570	250	2970	1170	770	320	1990	1160	770**	8960
	Median	690	0	1812	752	0	0	0	510	0	5032
	[IQR]	[690, 690]	[0, 0]	[1359, 3171]	[417, 1439]	[0, 0]	[0, 0]	[0, 356]	[68, 1496]	[0, 0]	[3089, 9061]
75–84	Mean	630	160	3900	1170	2290	300	5270	990	-	13180
	Median	690	0	2718	815	0	0	193	408	-	7099
	[IQR]	[690, 690]	[0, 0]	[1359, 4530]	[444, 1444]	[0, 0]	[0, 0]	[0, 4620]	[68, 1379]	-	[4072, 15453]
≥85	Mean	640	80	4090	860	3640	360	9290	580	-	15580
	Median	690	0	3171	653	0	0	121	102	-	7002
	[IQR]	[690, 690]	[0, 0]	[1925, 4983]	[409, 982]	[0, 3109]	[0, 0]	[0, 7192]	[0, 612]	-	[4126, 15985]
***Gender***												
Male	Mean	620	310	2810	940	700	500	1830	950	7280	11770
	Median	690	0	1359	629	0	0	0	340	3470	6056
	[IQR]	[690, 690]	[0, 0]	[906, 3171]	[330, 1156]	[0, 0]	[0, 0]	[0, 0]	[34, 1190]	[0, 15564]	[3203, 15864]
Female	Mean	620	140	3330	970	1640	350	4290	1150	3770	12620
	Median	690	0	2265	675	0	0	0	476	1596	7006
	[IQR]	[690, 690]	[0, 0]	[1359, 4077]	[380, 1134]	[0, 0]	[0, 0]	[0, 1716]	[68, 1598]	[0, 7271]	[3859, 14132]
***ISS***											
1–3	Mean	540	30	1490	860	60	210	2060	630	3910	7030
	Median	690	0	906	502	0	0	0	136	1596	3764
	[IQR]	[4, 690]	[0, 0]	[906, 1359]	[260, 1032]	[0, 0]	[0, 0]	[0, 0]	[0, 698]	[0, 5543]	[2229, 7418]
4–8	Mean	520	60	2640	930	770	330	1960	1040	6330	11170
	Median	690	0	1359	627	0	0	0	442	3392	6538
	[IQR]	[4, 690]	[0, 0]	[906, 2718]	[342, 1079]	[0, 0]	[0, 0]	[0, 303]	[68, 1385]	[0, 10853]	[3418, 14422]
9–15	Mean	650	200	3770	1020	2170	410	4750	1200	6220	14530
	Median	690	0	2718	705	0	0	0	566	3115	8203
	[IQR]	[690, 690]	[0, 0]	[1812, 4530]	[444, 1219]	[0, 0]	[0, 0]	[0, 1472]	[68, 1632]	[0, 10919]	[4694, 17291]
16+	Mean	1400	2290	7510	1160	2460	2090	1990	1740	8640	23750
	Median	690	2426	4077	934	0	0	0	787	7174	15292
	[IQR]	[690, 690]	[0, 3639]	[1812, 8154]	[403, 1537]	[0, 0]	[0, 0]	[0, 488]	[144, 2345]	[0, 16762]	[7338, 31316]

IQR Interquartile Range

*Costs of ED visit not included in table but included in calculation total costs as price is same for all respondents (€265)

**Productivity costs calculated for working population (18-67y), so only costs 65-67y included

### 3.3. Post-hospital medical costs

The average post-hospital medical costs were €5110 per patient, with homecare contributing the largest amount (€3070) followed by the costs of staying in an institution (€1140). In total, 5.9% of the patients stayed at a nursing home for on average 37 days, 4.6% stayed at a care home for on average 41 days and 6.4% stayed in a rehabilitation center for on average 41 days. The median costs and interquartile range show that costs of stay/day treatment in an institution and homecare in most subgroups are skewed, and the mean costs are driven by only a small group of respondents with extremely high costs. Post-hospital medical costs were on average €7190 for the group of elderly (65y+). Among this group of elderly homecare contributed most (68.6%) to the post-hospital medical costs. Furthermore, we found that costs of stay in an institution and homecare increased with age, whereas costs of day treatment fluctuated with age.

### 3.4. Productivity costs

Approximately one third (31.2%, n = 561) of the respondents within the working age range (18–67 years, n = 1797) indicated not to be in paid employment before sustaining injury, of which 88% was aged 45-67y. Within the group that was in paid employment before sustaining injury, 82.7% (n = 1022) indicated to have missed workdays due to injury, with a mean duration of 13.6 work weeks. Mean productivity costs (€5830) were higher than in-hospital costs and post-hospital costs for the working population (18-67y). Productivity costs were almost twice as high for males compared to females (males €7280 vs. females €3770). A larger percentage of males than females was in paid employment before injury (73.6% vs. 61.5%, p < 0.001), and mean number of hours work missed was higher for males than for females (514 vs. 389 hours, p = 0.003). Furthermore, productivity costs were higher for the younger working population (25–44 years) than for the older working population (45–67 years), with a mean of €7830 versus €5330 per person. Once returned to work, respondents in the working age (18–67 years) rated their productivity as 6 out of 10 (SD = 2.1, n = 607). The mean number of days at work with reduced productivity was 35 days (SD = 56.5, n = 581).

### 3.5. Total costs

**Fig 2:**
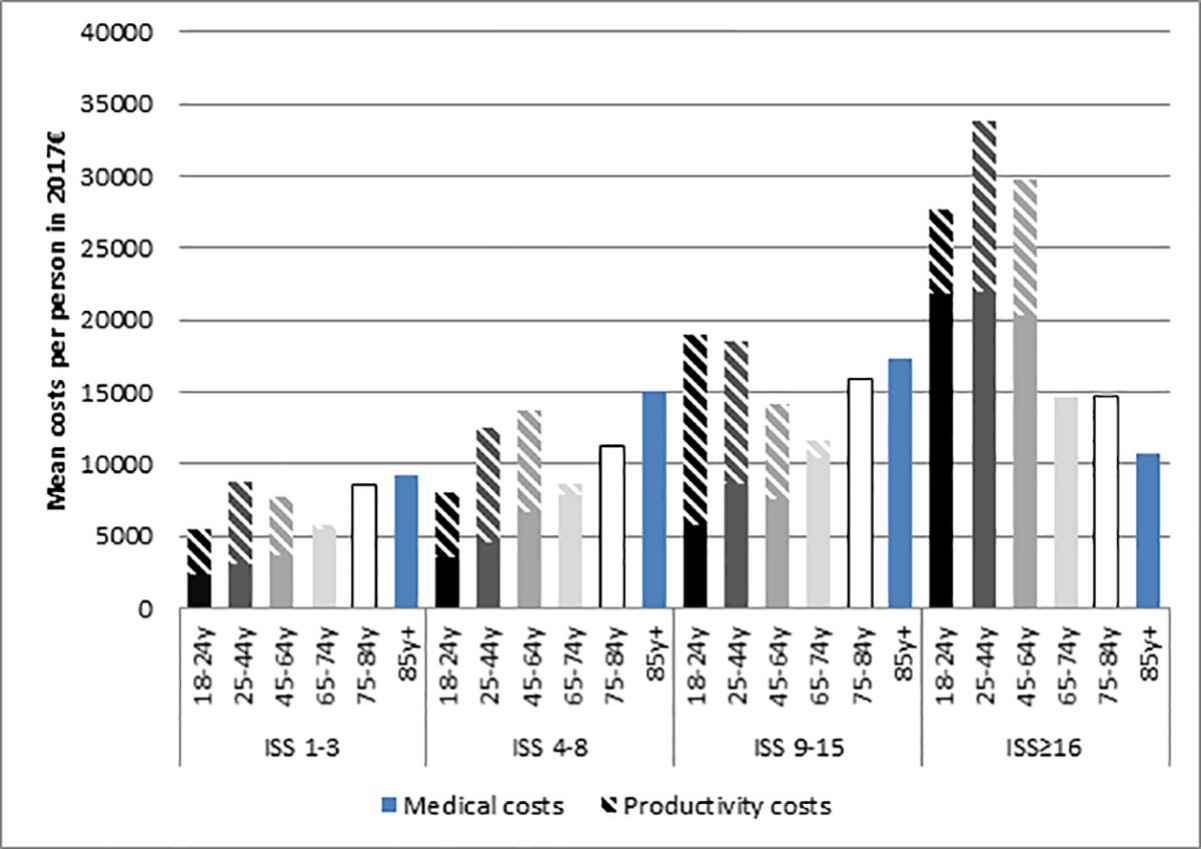
Mean total medical costs and productivity costs of injury per person, per age group per ISS category in 2017 €. Note: Productivity costs for age group 65–74 are only for age 65-67y.

In total, the average costs per patient were €12,190. No pattern was found in the total costs per person over age groups, as mean total costs were lowest for age 18-24y (€7980) and for age 65-74y (€8960). Due to the inclusion of productivity costs, and the absence of productivity costs after the age of 67, there is no clear pattern in total costs. Highest total costs per person were found for age 85 years and over, with a mean of €15,580. Fig 2 illustrates the fluctuation of costs over age groups. Comparing males to females, it was found that females had on average higher costs than males (females: €12,620 vs. males: €11,770).

### 3.6. Costs per ISS-category

Mean in-hospital costs per person increased with increasing injury severity, from €2890 for ISS 1–3 to €11,590 for ISS≥16. This difference in in-hospital costs was mainly driven by differences between ICU costs, which were almost 100 times higher for the ISS≥16 respondents (ISS 1–3: €30 vs. ISS≥16: €2290), and costs for stay at a ward, which were more than 5 times higher (ISS 1–3: €1490 vs. ISS≥16: €7510). Post-hospital medical costs also increased with increasing injury severity up to ISS 9–15, but were lower in ISS≥16 than in ISS 9–15. Costs of homecare were an exception, with second lowest costs for ISS≥16 (€1990), and highest costs for respondents with ISS 9–15 (€4750). As homecare is more common for elderly people, it should be noted that only 18% of respondents in ISS≥16 was aged 75+ years, whereas in ISS 9–15 this proportion was 49%. Furthermore, 67% of participants in ISS 9–15 suffered from hip fracture, and homecare is likely to be provided after a hip fracture. The main driver of post-hospital medical costs was homecare for ISS 1–15, and costs of stay in an institution for ISS≥16. Productivity costs were again highest for the most severely injured (ISS≥16: €8640), and lowest for the minor injuries (ISS 1–3: €3910). In ISS≥16 74% of the respondents was in the working age range (18-67y). The mean total costs of injury, including both medical and productivity costs, increased with injury severity, but fluctuated over age groups within the ISS categories (Fig 2). Even though post-hospital medical costs were higher in ISS 9–15 than in ISS≥16, high in-hospital and productivity costs in ISS≥16 caused total costs to increase with injury severity over all severity categories. However, mean medical costs per person were found to be increasing with both age and injury severity. Furthermore, the increase in medical costs with injury severity was larger in the younger age groups (18-64year) than in the older age groups (65+ year). In addition, an interaction term for age and injury severity was tested for statistical significance. It was found that the interaction was statistically significant for in-hospital costs and total costs, but not for post-hospital costs and productivity costs.

**Fig 3:**
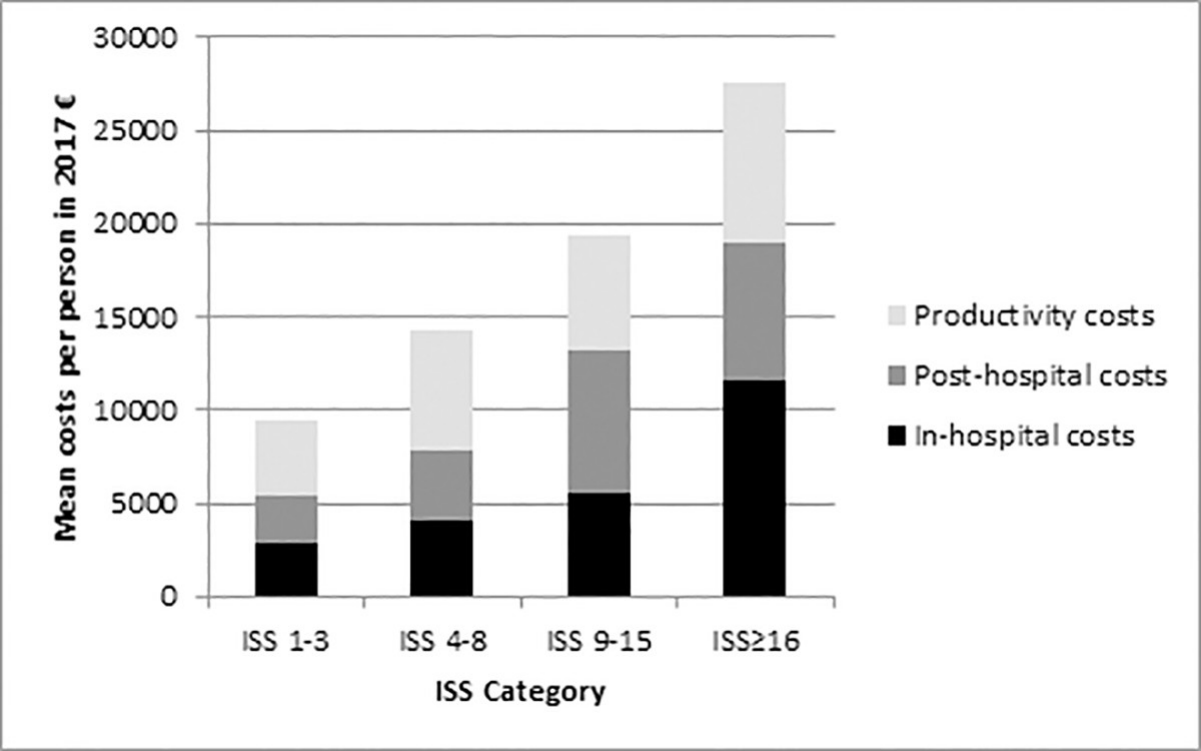
Total in-hospital, post-hospital medical and productivity costs per person, per ISS category in 2017 €.

Fig 3 shows the total costs per ISS category and per cost category. In-hospital costs increased with injury severity, whereas post-hospital medical costs were highest for ISS 9–15 (mean €7530 per person). Productivity costs were lowest for ISS 1–3 and highest for ISS≥16. In ISS 1–3 and ISS 4–8 approximately 45% of the total costs are caused by productivity costs, whereas in ISS 9–15 and ISS≥16 this is approximately one third.

## Discussion

### 4.1. Main findings

This study described the medical costs and productivity costs after trauma in a large multicentre prospective injury cohort study with varying injury severity. Mean total costs per person of injury, including both medical and productivity costs, increased with injury severity, but varied across age groups. However, mean medical costs per person increased with age. The difference in patterns across age groups for total costs and medical costs can be explained by the absence of productivity costs in age groups of 67+ year, and high productivity costs in younger age groups. Furthermore, an interaction term of age and injury severity was found to be significant for in-hospital costs, which might affect the pattern of medical costs. In-hospital costs were also found to increase with injury severity, mainly driven by costs of stay at a ward. Furthermore, in-hospital costs were the smallest cost component for injury severity categories 1–3 and 9–15; however, for ISS≥16 it was the largest cost component. Post-hospital medical costs increased with injury severity up to ISS 9–15, and were mainly driven by costs of homecare. ISS≥16 was an exception, where post-hospital medical costs were lower than in ISS 9–15 and were not driven by costs of homecare, which can be explained by the small percentage of elderly in this injury severity category (18% vs. 49% in ISS9-15). Productivity costs were increasing with injury severity, except for ISS 9–15, due to the large proportion of elderly (67y+) in this group that have no productivity costs. In ISS 1–3 and ISS 4–8 productivity costs comprised a large part of total costs of injury. Productivity costs were highest in all ISS categories for age group 24–44, except for ISS 9–15. After return to work, productivity level was just over half, meaning that productivity costs are actually even higher.

All costs were reported as mean values, with the median and interquartile range available as well. The median and interquartile range show that costs of ICU stay, stay in an institution, day treatment and homecare are skewed, meaning that outliers affect the mean costs. This can be seen in mean costs for ICU, as the IQR is zero for the total population, while subgroup ISS≥16 has an IQR ranging from 0 to €3639. Furthermore, mean costs of homecare were affected by some outliers in the elderly population.

### 4.2. Comparison to previous studies

Costs of injury have been studied before, but not with a specific injury severity classification in combination with a detailed level of costs. The findings of the studies by Polinder et al. and Meerding et al. [3, 8] were in line with the findings of our study, as both studies found that (in-)hospital costs of patients (65y+) were rising with age. However, considering in-hospital costs, post-hospital medical costs and productivity costs together, mean total costs per person were found to be fluctuating over age in our research. This was due to the inclusion of productivity costs in the working age population, and the absence of productivity costs at older age.

Another study by Polinder et al. [28] pointed out that high medical costs in especially elderly women were mainly caused by hip injuries, which require expensive treatment. Hip fractures as single injury have an ISS of 9, meaning that this high cost group is included in ISS category 9–15 [29]. As 67% of the injuries in the ISS 9–15 category were elderly patients with a hip fracture, this explains why some of the health care costs in this group are extremely high compared to other ISS categories, for example costs of homecare.

### 4.3. Strengths and limitations

This study had several strengths and limitations. A major strength of our study was that the in-hospital, post-hospital medical costs and productivity costs were calculated with a high level of detail. The in-hospital costs included costs of diagnostics, which is unique for a study of injury costs in a general injury population. Furthermore, because ISS of the respondents was available, we could calculate each of the cost components by injury severity level, which has not been studied before.

A limitation of our study was that the rate of respondents that completed follow-up up to 24 months post-injury was relatively low. A possible cause of the low response rate was the length of the survey, which included measurement instruments on health status, psychological consequences, health care consumption, work absenteeism and work presenteeism. Patients with a rapid recovery are likely to have been lost to follow-up sooner, as questions on health status, psychological consequences, health care consumption and work might seem irrelevant to them when recovery took place shortly after their injury. This could have resulted in a participation bias of the sample that filled out more questionnaires and therefore an overestimation of the actual mean costs. This affects both internal validity of our findings and generalizability of the results to the Dutch injury population. In addition, it was found that demographics of responders and non-responders differed with respect to a number of characteristics, which supports also the expectation of non-response bias.

A second limitation of this study was that several assumptions had to be made to calculate the costs of injury. As not all respondents filled out the questionnaires completely, the number of respondents that was included in the cost calculations of different items varied. Even though it is a limitation that the group of respondents is not the same for each cost item, the inclusion of all available responses enables the use of all available information. Another assumption that should be taken into account is the duration between different surveys. It is likely that responses one month after injury (informing on the previous month), 3 months after injury (informing on the previous 2 months) and 6 months after injury (informing on the previous 3 months) are more accurate than responses 12 months after injury (informing on the previous 6 months) and 24 months after injury (informing on the previous 12 months), considering recall bias. Furthermore, to enable total cost calculations, zero-values had to be imputed for missing values, which may have led to an underestimation of total cost.

A third limitation of the study was the inability to control for the deviation between injury related health care and health care that was not related to the injury. Costs of homecare in particular were found to be high in ISS1-3, indicating that reported homecare is not only injury related. Even though respondents were asked to report only injury related care activities, the results indicate that also homecare related to, for example, comorbidities was reported. Therefore, the costs of homecare and post-hospital medical costs are most likely an overestimation of injury related costs, especially in the older age groups.

A final limitation of our study was that for the calculation of productivity costs the average wage per hour was used independently of education level. General average wage for male and female was available only, with no subdivision for education level. As the percentage of respondents with a low education level in our study (52.4%) was much higher than the percentage for the Dutch working population (28.6%) [30], this may have led to an overestimation of the true productivity costs. Furthermore, only 62% of the respondents in the working age population indicated to be in paid employment before injury, which could be related to relatively low education level of the population. With an extremely high percentage of non-working respondents in the working age range, this could lead to an underestimation of productivity costs, as productivity costs are zero for this group.

### 4.4. Implications for practice

The findings of this study showed that productivity costs were high. With a long duration of work absence, and on average just over half the productivity level at work on return, an intervention dedicated to improve return to work and productivity level after injury might reduce the total costs of injury. Furthermore, it was shown that costs of injury are increasing with severity, but fluctuated over age groups. With the group of 85y+ having the highest costs of injury, this is a relevant group for injury prevention, as injuries in this age group often result from falls and programs directed to prevent falls are available.

### 4.5. Conclusions

In conclusion, medical costs of injury were increasing with injury severity. The main contributor to in-hospital costs are the costs of stay at a ward, and for post-hospital costs the costs of homecare, except for ISS≥16. Productivity costs comprised a large part of the total costs of injury for the working age population (18-67y) in all severity categories, indicating that these costs should be included in the analyses of injury costs as well, and should be a main focus point of interventions aiming to reduce costs of injury. Total medical costs were found to be highest for the oldest age group (85y+), indicating that among this age group injury prevention is especially important.

## Supporting information

S1 Table Unit costs (2017€). (DOCX)

S2 Table Mean health care costs and productivity costs in 2017 euro per ISS category, gender and age group for responders; including range and number of respondents. (DOCX)
