# Supplementary material for: Medical and productivity costs after trauma
Source: PLoS One. 2019 Dec 30;14(12):e0227131. doi: 10.1371/journal.pone.0227131 (PMC6936839; doi:10.1371/journal.pone.0227131)
Supplement: S1 Table — (DOCX) [file pone.0227131.s001.docx]

**S1 Table**

Table S1: Unit costs (2017 €)

| Resource | Unit costs |
| --- | --- |
| *Transport* |  |
| Self-transport | €4.43/ride |
| Ambulance transport | €690/ride |
| Ambulance transport with support of air medical services | €5831 |
| Ambulance transport with support of ground medical services | €5831 |
| Trauma helicopter transport | €5281 |
| *Hospital* |  |
| Visit ED | €265/visit |
| In-hospital day generic hospital | €453/day |
| In-hospital day ICU | €1213/day |
| Day-treatment hospital | €282/day |
| Outpatient clinic general hospital | €82/visit |
| *Long term care* |  |
| Nursing home | €172/day, €69/part of day care |
| Rehabilitation | €471/day, €157/hour day care |
| Psychiatric institution | €309/day, €100/day care |
| *Home care* |  |
| Domestic care | €22/hour |
| Help with all day activities | €55/hour |
| Nursing | €75/hour |
| *Health care provider* |  |
| General practitioner (GP) | €34/visit |
| Company doctor | €53/visit |
| Psychologist | €65/visit |
| Social worker | €67/visit |
| Physiotherapist | €34/visit |
| Occupational therapist | €34/visit |
| Speech therapist | €31/visit |
| Dietician | €21/visit |
| *Productivity* |  |
| Paid work male | €38.78/hour |
| Paid work female | €32.33/hour |
