# Supplementary material for: Medical and productivity costs after trauma
Source: PLoS One. 2019 Dec 30;14(12):e0227131. doi: 10.1371/journal.pone.0227131 (PMC6936839; doi:10.1371/journal.pone.0227131)
Supplement: S2 Table — (DOCX) [file pone.0227131.s002.docx]

**S2 Table**

Table S2: Mean health care costs and productivity costs in 2017 euro per ISS category, gender and age group for responders; including range and number of respondents

|  |  | | In-hospital* | | | | | | | | Post-hospital | | | | | | | | | Productivity | | Total | |
| --- | --- | --- | --- | --- | --- | --- | --- | --- | --- | --- | --- | --- | --- | --- | --- | --- | --- | --- | --- | --- | --- | --- | --- |
|  | |  | Ambulance transport | | ICU | | Ward | | Diagnostics | | Stay in institution | | Day-treatment | | Home-care | | Practitioner visit | | | Productivity costs** | | Total costs | |
| Total | Mean | | 620 | | 220 | | 3070 | | 950 | | 1140 | | 430 | | 3070 | | 1050 | | | 5830 | | 12190 | |
|  | Median | | 690 | | 0 | | 1812 | | 649 | | 0 | | 0 | | 0 | | 407 | | | 2654 | | 6570 | |
|  | [IQR] | | [690, 690] | | [0, 0] | | [906, 3624] | | [357, 1143] | | [0, 0] | | [0, 0] | | [0, 495] | | [52, 1368] | | | [0, 10078] | | [3551, 14786] | |
|  | [Range] | | [4, 5831] | | [0, 6065] | | [453, 77010] | | [0, 43613] | | [0, 55107] | | [0, 34854] | | [0, 356872] | | [0, 16373] | | | [0, 33257] | | [722, 368498] | |
|  | n | | 3687 | | 3521 | | 3506 | | 2602 | | 2946 | | 3150 | | 3339 | | 3342 | | | 1593 | | 3521 | |
| *Age* |  | |  | |  | |  | |  | |  | |  | |  | |  | | |  | |  | |
| 18-24 | Mean | | 720 | | 410 | | 1920 | | 520 | | 230 | | 400 | | 460 | | 890 | | | 4940 | | 7980 | |
|  | Median | | 690 | | 0 | | 906 | | 383 | | 0 | | 0 | | 0 | | 170 | | | 2399 | | 3844 | |
|  | [IQR] | | [347, 690] | | [0, 0] | | [453, 1359] | | [225, 687] | | [0, 0] | | [0, 0] | | [0, 0] | | [0, 901] | | | [165, 8209] | | [1916, 11301] | |
|  | [Range] | | [4, 5831] | | [0, 6065] | | [453, 31257] | | [38, 3073] | | [0, 24021] | | [0, 12560] | | [0, 45760] | | [0, 10221] | | | [0, 18624] | | [760, 91087] | |
|  | n | | 153 | | 148 | | 147 | | 94 | | 136 | | 116 | | 123 | | 123 | | | 89 | | 148 | |
| 25-44 | Mean | | 650 | | 310 | | 2230 | | 640 | | 420 | | 640 | | 760 | | 1030 | | | 7830 | | 12310 | |
|  | Median | | 690 | | 0 | | 1359 | | 495 | | 0 | | 0 | | 0 | | 325 | | | 5792 | | 7360 | |
|  | [IQR] | | [690, 690] | | [0, 0] | | [906, 2265] | | [279, 832] | | [0, 0] | | [0, 0] | | [0, 0] | | [60, 1274] | | | [1596, 13460] | | [3145, 16802] | |
|  | [Range] | | [4, 5831] | | [0, 6065] | | [453, 35334] | | [0, 6242] | | [0, 25905] | | [0, 22608] | | [0, 148554] | | [0, 16373] | | | [0, 32592] | | [722, 162926] | |
|  | n | | 421 | | 402 | | 400 | | 275 | | 372 | | 356 | | 381 | | 381 | | | 334 | | 402 | |
| 45-64 | Mean | | 610 | | 260 | | 2570 | | 860 | | 470 | | 530 | | 1000 | | 1230 | | | 6410 | | 12650 | |
|  | Median | | 690 | | 0 | | 1359 | | 567 | | 0 | | 0 | | 0 | | 572 | | | 3691 | | 8285 | |
|  | [IQR] | | [690, 690] | | [0, 0] | | [906, 2718] | | [320, 1000] | | [0, 0] | | [0, 0] | | [0, 0] | | [102, 1663] | | | [0, 11047] | | [3694, 17363] | |
|  | [Range] | | [4, 5831] | | [0, 6065] | | [453, 62967] | | [38, 10753] | | [0, 42390] | | [0, 34854] | | [0, 86152] | | [0, 12681] | | | [0, 33257] | | [722, 98563] | |
|  | n | | 1100 | | 1061 | | 1056 | | 750 | | 988 | | 1001 | | 1037 | | 1037 | | | 947 | | 1061 | |
| 65-74 | Mean | | 570 | | 250 | | 2970 | | 1170 | | 770 | | 320 | | 1990 | | 1160 | | | 770** | | 8960 | |
|  | Median | | 690 | | 0 | | 1812 | | 752 | | 0 | | 0 | | 0 | | 510 | | | 0 | | 5032 | |
|  | [IQR] | | [690, 690] | | [0, 0] | | [1359, 3171] | | [417, 1439] | | [0, 0] | | [0, 0] | | [0, 356] | | [68, 1496] | | | [0, 0] | | [3089, 9061] | |
|  | [Range] | | [4, 5831] | | [0, 6065] | | [453, 77010] | | [38, 14767] | | [0, 55107] | | [0, 18184] | | [0, 91660] | | [0, 11608] | | | [0, 32592] | | [722, 116692] | |
|  | n | | 735 | | 699 | | 694 | | 514 | | 649 | | 673 | | 696 | | 695 | | | 223 | | 699 | |
| 75-84 | Mean | | 630 | | 160 | | 3900 | | 1170 | | 2290 | | 300 | | 5270 | | 990 | | | - | | 13180 | |
|  | Median | | 690 | | 0 | | 2718 | | 815 | | 0 | | 0 | | 193 | | 408 | | | - | | 7099 | |
|  | [IQR] | | [690, 690] | | [0, 0] | | [1359, 4530] | | [444, 1444] | | [0, 0] | | [0, 0] | | [0, 4620] | | [68, 1379] | | | - | | [4072, 15453] | |
|  | [Range] | | [4, 5831] | | [0, 4852] | | [453, 34881] | | [39, 43613] | | [0, 54636] | | [0, 15386] | | [0, 125044] | | [0, 11928] | | | - | | [722, 136912] | |
|  | n | | 761 | | 726 | | 725 | | 586 | | 527 | | 638 | | 685 | | 683 | | | - | | 726 | |
| ≥85 | Mean | | 640 | | 80 | | 4090 | | 860 | | 3640 | | 360 | | 9290 | | 580 | | | - | | 15580 | |
|  | Median | | 690 | | 0 | | 3171 | | 653 | | 0 | | 0 | | 121 | | 102 | | | - | | 7002 | |
|  | [IQR] | | [690, 690] | | [0, 0] | | [1925, 4983] | | [409, 982] | | [0, 3109] | | [0, 0] | | [0, 7192] | | [0, 612] | | | - | | [4126, 15985] | |
|  | [Range] | | [4, 690] | | [0, 3639] | | [906, 57531] | | [40, 6969] | | [0, 52752] | | [0, 20724] | | [0, 356872] | | [0, 14135] | | | - | | [1175, 368498] | |
|  | n | | 517 | | 485 | | 484 | | 383 | | 274 | | 366 | | 417 | | 423 | | | - | | 485 | |
| *Gender* | | |  | |  | |  | |  | |  | |  | |  | |  | | |  | |  | |
| Male | | Mean | 620 | | 310 | | 2810 | | 940 | | 700 | | 500 | | 1830 | | 950 | | | 7280 | | 11770 | |
|  |  | Median | 690 | | 0 | | 1359 | | 629 | | 0 | | 0 | | 0 | | 340 | | | 3470 | | 6056 | |
|  |  | [IQR] | [690, 690] | | [0, 0] | | [906, 3171] | | [330, 1156] | | [0, 0] | | [0, 0] | | [0, 0] | | [34, 1190] | | | [0, 15564] | | [3203, 15864] | |
|  |  | [Range] | [4, 5831] | | [0, 6065] | | [453, 77010] | | [38, 43613] | | [0, 54636] | | [0, 34854] | | [0, 162448] | | [0, 16373] | | | [0, 33257] | | [722, 171025] | |
|  |  | n | 1827 | | 1752 | | 1742 | | 1249 | | 1571 | | 1580 | | 1658 | | 1658 | | | 936 | | 1752 | |
| Female | | Mean | 620 | | 140 | | 3330 | | 970 | | 1640 | | 350 | | 4290 | | 1150 | | | 3770 | | 12620 | |
|  |  | Median | 690 | | 0 | | 2265 | | 675 | | 0 | | 0 | | 0 | | 476 | | | 1596 | | 7006 | |
|  |  | [IQR] | [690, 690] | | [0, 0] | | [1359, 4077] | | [380, 1134] | | [0, 0] | | [0, 0] | | [0, 1716] | | [68, 1598] | | | [0, 7271] | | [3859, 14132] | |
|  |  | [Range] | [4, 5831] | | [0, 6065] | | [453, 57531] | | [0, 14767] | | [0, 55107] | | [0, 22608] | | [0, 356872] | | [0, 14135] | | | [0, 23256] | | [722, 368498] | |
|  |  | n | 1860 | | 1769 | | 1764 | | 1353 | | 1375 | | 1570 | | 1681 | | 1684 | | | 657 | | 1769 | |
| *ISS* | |  |  | |  | |  | |  | |  | |  | |  | |  | | |  | |  | |
| 1-3 | | Mean | 540 | | 30 | | 1490 | | 860 | | 60 | | 210 | | 2060 | | | 630 | | 3910 | | 7030 | |
|  |  | Median | 690 | | 0 | | 906 | | 502 | | 0 | | 0 | | 0 | | | 136 | | 1596 | | 3764 | |
|  |  | [IQR] | [4, 690] | | [0, 0] | | [906, 1359] | | [260, 1032] | | [0, 0] | | [0, 0] | | [0, 0] | | | [0, 698] | | [0, 5543] | | [2229, 7418 | |
|  |  | [Range] | [4, 5831] | | [0, 6065] | | [453, 32616] | | [38, 10753] | | [0, 10836] | | [0, 20096] | | [0, 91660] | | | [0, 12675] | | [0, 22349] | | [722, 99112] | |
|  |  | n | 854 | | 793 | | 793 | | 604 | | 774 | | 714 | | 745 | | | 742 | | 406 | | 793 | |
| 4-8 | | Mean | 520 | | 60 | | 2640 | | 930 | | 770 | | 330 | | 1960 | | | 1040 | | 6330 | | 11170 | |
|  |  | Median | 690 | | 0 | | 1359 | | 627 | | 0 | | 0 | | 0 | | | 442 | | 3392 | | 6538 | |
|  |  | [IQR] | [4, 690] | | [0, 0] | | [906, 2718] | | [342, 1079] | | [0, 0] | | [0, 0] | | [0, 303] | | | [68, 1385] | | [0, 10853] | | [3418, 14422] | |
|  |  | [Range] | [4, 5831] | | [0, 4852] | | [453, 57531] | | [39, 14767] | | [0, 55107] | | [0, 20724] | | [0, 115400] | | | [0, 16373] | | [0, 32592] | | [722, 130686] | |
|  |  | n | 1212 | | 1160 | | 1155 | | 881 | | 1026 | | 1075 | | 1127 | | | 1129 | | 648 | | 1160 | |
| 9-15 | | Mean | | 650 | | 200 | | 3770 | | 1020 | | 2170 | | 410 | | 4750 | | | 1200 | | 6220 | | 14530 |
|  |  | Median | | 690 | | 0 | | 2718 | | 705 | | 0 | | 0 | | 0 | | | 566 | | 3115 | | 8203 |
|  |  | [IQR] | | [690, 690] | | [0, 0] | | [1812, 4530] | | [444, 1219] | | [0, 0] | | [0, 0] | | [0, 1472] | | | [68, 1632] | | [0, 10919] | | [4694, 17291] |
|  |  | [Range] | | [4, 5831] | | [0, 6065] | | [453, 77010] | | [0, 43613] | | [0, 54636] | | [0, 16146] | | [0, 356872] | | | [0, 14135] | | [0, 33257] | | [722, 368498] |
|  |  | n | | 1426 | | 1378 | | 1373 | | 1050 | | 1000 | | 1177 | | 1269 | | | 1273 | | 422 | | 1378 |
| 16+ | | Mean | | 1400 | | 2290 | | 7510 | | 1160 | | 2460 | | 2090 | | 1990 | | | 1740 | | 8640 | | 23750 |
|  |  | Median | | 690 | | 2426 | | 4077 | | 934 | | 0 | | 0 | | 0 | | | 787 | | 7174 | | 15292 |
|  |  | [IQR] | | [690, 690] | | [0, 3639] | | [1812, 8154] | | [403, 1537] | | [0, 0] | | [0, 0] | | [0, 488] | | | [144, 2345] | | [0, 16762] | | [7338, 31316] |
|  |  | [Range] | | [4, 5831] | | [0, 6065] | | [453, 62967] | | [44, 5211] | | [0, 42390] | | [0, 34854] | | [0, 63830] | | | [0, 10221] | | [0, 30264] | | [1504, 98563] |
|  |  | n | | 187 | | 184 | | 179 | | 50 | | 142 | | 159 | | 172 | | | 172 | | 106 | | 184 |

*Costs of ED visit not included in table but included in calculation total costs as price is same for all respondents (€265)
**Productivity costs calculated for working population (18-67y)
